# Biochemical and Molecular Insights into Variation in Sesame Seed Antioxidant Capability as Revealed by Metabolomics and Transcriptomics Analysis

**DOI:** 10.3390/antiox13050514

**Published:** 2024-04-25

**Authors:** Senouwa Segla Koffi Dossou, Zishu Luo, Qianchun Deng, Rong Zhou, Yanxin Zhang, Donghua Li, Huan Li, Koffi Tozo, Jun You, Linhai Wang

**Affiliations:** 1 Key Laboratory of Biology and Genetic Improvement of Oil Crops of the Ministry of Agriculture and Rural Affairs, Oil Crops Research Institute of the Chinese Academy of Agricultural Sciences, Wuhan 430062, China; 2 Laboratory of Plant Biotechnology and Physiology, University of Lomé, Lomé 01 BP 1515, Togo; koffitozo@gmail.com

**Keywords:** sesame, antioxidant, polyphenol profiling, transcriptomics, seed coat color, LC-MS

## Abstract

Sesame seeds are important resources for relieving oxidation stress-related diseases. Although a significant variation in seeds’ antioxidant capability is observed, the underlying biochemical and molecular basis remains elusive. Thus, this study aimed to reveal major seed components and key molecular mechanisms that drive the variability of seeds’ antioxidant activity (AOA) using a panel of 400 sesame accessions. The seeds’ AOA, total flavonoid, and phenolic contents varied from 2.03 to 78.5%, 0.072 to 3.104 mg CAE/g, and 2.717 to 21.98 mg GAE/g, respectively. Analyses revealed that flavonoids and phenolic acids are the main contributors to seeds’ AOA variation, irrespective of seed coat color. LC-MS-based polyphenol profiling of high (HA) and low (LA) antioxidant seeds uncovered 320 differentially accumulated phenolic compounds (DAPs), including 311 up-regulated in HA seeds. Tricin, persicoside, 5,7,4′,5′-tetrahydro-3′,6-dimethoxyflavone, 8-methoxyapigenin, and 6,7,8-tetrahydroxy-5-methoxyflavone were the top five up-regulated in HA. Comparative transcriptome analysis at three seed developmental stages identified 627~2357 DEGs and unveiled that differential regulation of flavonoid biosynthesis, phenylpropanoid biosynthesis, and stilbene biosynthesis were the key underlying mechanisms of seed antioxidant capacity variation. Major differentially regulated phenylpropanoid structural genes and transcription factors were identified. *SINPZ0000571* (MYB), *SINPZ0401118* (NAC), and *SINPZ0500871* (C3H) were the most highly induced TFs in HA. Our findings may enhance quality breeding.

## 1. Introduction

Sesame (*Sesamum indicum* L.) is a vital industrial and oilseed crop in the Pedaliaceae family [1]. Its high medicinal and nutritional values have raised interest in sesame products’ consumption worldwide and use in various industries, including pharmaceutics, foods, biodiesel, cosmetics, etc. [2,3]. Sesame seeds are abounding in nutraceuticals, including essential fatty acids, inherent lignans (sesamin, sesamolin, sesaminol, sesamol, sesamolinol, etc.), tocopherols, melatonin, phytosterols, and other essential antioxidants [3,4,5,6]. Accordingly, sesame seed consumption is associated with enormous health benefits. For instance, clinical trials and in vivo and in vitro investigations showed that they have antioxidant, anti-diabetes, anti-hyperlipidemia, kidney and liver protection, anti-inflammatory, cardiovascular protection, anti-hypertension, antitumor, and anti-cancer properties [3,4,5,7]. The high-antioxidant capacity of sesame seeds has led them to be used to improve the stability and quality and prevent autoxidation of countless foodstuffs [8,9]. Unfortunately, although there is evidence of great variation in sesame seeds’ antioxidant capacity, the underlying biochemical and molecular bases remain poorly elucidated, limiting efforts to develop novel sesame varieties with improved medicinal potentials.

Diverse factors, such as origin, seed coat color, and growing and processing conditions, impact the antioxidant capacity of sesame seeds [10,11,12]. Early studies have demonstrated that black sesame seeds possess higher antioxidative ability than other colored seeds [10,13,14]. However, these studies analyzed a small number of varieties, so it would be more appropriate to explore a vast population comprised high numbers of different colored sesame seeds before making a statement. Moreover, the huge beneficial health effects of sesame-specific lignans, including sesamin, sesamol, sesamolin, sesaminol, etc., result in the association of seeds’ antioxidant activity variation to the difference in lignan content only [5,15,16]. Nevertheless, some sesame seeds with low lignan content have shown higher antioxidant activities than those with high lignan content [10,17,18]. Therefore, we hypothesized that changes in the composition of other antioxidants in the seeds may be the cause of the observed variability in antioxidant capacity.

A comparative analysis of the global metabolome of brown, white, black, and yellow sesame seeds disclosed that difference in the relative content of some phenolic compounds was the main causative factor of their antioxidant activity differences [14]. We inferred that considerable differences might exist between the polyphenol profiles of low and high-antioxidant sesame seeds. Polyphenols, including flavonoids (flavanols, anthocyanidins, flavonols, flavanones, flavones, and chalcones), stilbene, tannins, phenolic acids, and saponin are members of plant secondary metabolites [19,20]. They are excellent antioxidants and their daily consumption may lead to the relief of oxidative stress and the prevention of aging and several chronic and lifestyle diseases [19,20,21,22,23]. Among the oilseed crops, sunflower, soybean, brassica, and olive have received more attention than sesame in terms of polyphenol composition [24,25]. Therefore, a comprehensive characterization of polyphenol profile differences between HA and LA sesame seeds is of immense interest. It will enable the biochemical understanding of sesame seeds’ antioxidant capability variation and essential resources for gene-metabolite network analyses that may serve in quality breeding.

Phenylpropanoids are the most diverse class of natural products, regrouping phenolic acids, anthocyanins, flavonoids, monolignols, lignans, coumarins, and tannins [26]. These secondary metabolites perform tremendous functions in plants, such as nutrient uptake, photosynthesis, growth regulation, maintenance of redox homeostasis, and responses to biotic and abiotic stresses [26,27]. Their biosynthesis occurs from phenylalanine and tyrosine and is regulated by many transcription factors, among which MYB, NAC, and bHLH play critical roles [27,28,29,30]. The phenylpropanoid pathway involves key structural genes such as phenylalanine ammonia-lyase, cinnamate 4-hydroxylase, 4-coumarate-CoA ligase, chalcone synthase, flavonoid 3′-hydroxylase, chalcone isomerase, flavanone 3-hydroxylase, UDP-glucose:flavonoid-3-Oglucosyl-transferase, etc. [27,28,29]. Identifying key differentially regulated genes in this pathway between HA and LA may offer a great opportunity to modulate the polyphenol profile of sesame seeds.

In this study, we analyzed the AOA (antioxidant activity), TFC (total flavonoid content), and TPC (total phenolic content) of 400 sesame accessions. Furthermore, we conducted UPLC_MS/MS (ultra-performance liquid chromatography-mass spectroscopy)-based widely targeted polyphenol profiling transcriptome analysis of HA and LA seeds. Our objectives were to identify major seed components governing variation in sesame seed antioxidant capacity and achieve insight into the associated molecular mechanisms. The results of this study provide biochemical and genetic indicators for the improvement in sesame seed antioxidants’ composition.

## 2. Materials and Methods

### 2.1. Plant Materials and Growing Conditions

Four hundred sesame accessions were analyzed in this study (Supplementary Table S1). They were given by the Oil Crops Research Institute of the Chinese Academy of Agricultural Sciences (OCRI-CAAS), Wuhan, China. The sesame population included 45, 85, 32, and 234 black, brown, yellow, and white seeds, respectively. All seeds were cultivated under the same environmental conditions in Wuhan, China. All required agronomic practices for sesame were applied equally [3]. During harvesting in September 2022, seed samples were collected in triplicate for all genotypes. Each replicate was a mixture of seeds from twelve individual plants. The seed samples were stored in the OCRI seeds room until the evaluation of the AOA, TPC, and TFC evaluations.

For the comparative polyphenol profiling and transcriptomics analysis, three high (HA; NS089, NS287, and NS148) and three low (LA; NS100, NS009, and NS120) antioxidant varieties were selected and cultivated similarly from June to September 2023 (Table S1). Developing seeds of NS089 (HA) NS100 (LA) were sampled at 10, 20, and 30 DPA (days post-anthesis) for transcriptome sequencing. Seed samples of the six varieties were prepared (three replications) after harvest for the metabolomics analysis. All samples were directly frozen in liquid nitrogen and kept at −80 °C until used.

### 2.2. Assessment of Total Phenolic (TPC) and Flavonoid (TFC) Contents and Antioxidant Activity (AOA)

Seed extraction for AOA, TPC, and TFC evaluation was achieved following previously described methods [31,32]. Briefly, for each replicate, 0.5 g of seeds were extracted for 4 h (constant shaking in darkness) with 5 mL 80% ethanol. Next, centrifugation (5000× *g*, 12 min) was followed by supernatant collection separately. All seed extracts were stored at −20 °C during the analyses. 

The TFC and TPC were analyzed according to the methods of Choi et al. [32]. Regarding the TPC, 400 μL dH_2_O and 100 μL Folin-Ciocalten reagent were added to 100 μL of seed extract, mixed well, and left for 6 min. Thereafter, 1 mL of Na_2_CO_3_ (7% *m*/*v*) and 800 μL of dH_2_O were added subsequently. After 90 min of reaction at room temperature, the absorbance of the mixture was recorded at 760 nm (UV5200, Shanghai Metash Instruments Co., Ltd., Shanghai, China). In the blank, 80% ethanol was used in place of the extract. The TPC values were expressed as mg GAE/g (gallic acid equivalent per gram) of seeds (y = 1.971x − 0.0068, R^2^ = 0.99).

Regarding the TFC, 1 mL of seed extract was mixed with 150 μL of NaNO_2_ (5% *m*/*v*) and the mixture was kept for 6 min. Thereafter, 300 μL of AlCl_3_·6H_2_O (10% *m*/*v*) was added, followed by 1 mL of 1 M NaOH another 6 min later. Finally, 1.05 mL dH_2_O was added, followed by absorbance at 510 nm fifteen minutes later. The TFC was estimated using y = 3.253x + 0.1447 (R^2^ = 0.9702) and expressed as mg CAE/g (catechin equivalent per gram) seeds. The AOA of the seeds was evaluated via DPPH assays, as recently reported [14,33].

### 2.3. Polyphenol Extraction and UPLC-MS/MS Analysis

Seeds were freeze-dried and reduced to powder using a mixer mill (MM 400, Retsch, Haan, Germany). The crushing was operated at 30 Hz for 1.5 min. Next, 100 mg of each sample was extracted at 4 °C (overnight) with 1.2 mL of 70% methanol. After centrifugation (20 min at 12,000× *g*), the supernatants were collected and filtrated through a micropore membrane (0.22 μm, SCAA-104, ANPEL, Shanghai, China). The extracts were stored at −20 °C up to the UPLC-ESI-QqQLIT-MS/MS analysis at Metware Biotechnology Co., Ltd., (MWDB), Wuhan, China [14,34,35,36]. Equal volumes of all sample seed extracts were mixed to constitute QC (quality control) samples. The metabolomics was performed as per previously described methods [14,35,36]. The liquid phase and MS conditions are detailed in Table S2.

### 2.4. Identification and Quantification of Phenolic Compounds

The spectrum information, mass spectra, and retention times were integrated to qualitatively identify the phenolic compounds. Specifically, the values of Q1 (precursor ions) and Q3 (product ion), retention times, fragmentation patterns, collision energy, and de-clustering potential were allied with standards when available (Sigma-Aldrich, St. Louis, MO, USA). When no standards were available, the compounds were structurally confirmed via the MWDB self-build database and verification in open databases (KNApSAcK, MassBank, MoTo DB, HMDB, and METLIN) [34,35]. The relative contents of the identified polyphenols were calculated via the triple quadrupole (QqQ) MS analysis (MRM modes) using the integrated SCIEX-OS software (version 1.4). 

### 2.5. Data Analysis

All multivariate analyses were achieved in R (version 3.5.0) after quality validation and subsequent standardization of the data. The statistical packages pheatmap, MetaboAnalystR, cor, and prcomp were used for hierarchical clustering analysis, orthogonal partial least squares discriminant analysis, correlation analysis, and principal component analysis. The variable importance of the projection (VIP) value of the phenolic compounds was extracted from the OPLS-DA results. Differentially accumulated metabolites (DAMs) were sorted out using the R-programming language ggplot2 program at thresholds of Log2FC ˃ 1, *p*-value < 0.05, and VIP ≥ 1. KEGG functional analysis of DAMs was carried out by mapping http://www.kegg.jp/kegg/pathway.html (accessed on 17 November 2023) and subsequent metabolite sets for enrichment analysis. Excel 2021 software and GraphPad Prism (v9.0.01, La Jolla, CA, USA) were used for data processing and graph construction. SRplot was also used for PCA and correlation analyses [37]. An ANOVA (analysis of variance) test was performed for multiple comparisons at *p* < 0.05.

### 2.6. RNA Extraction, Library Construction, Sequencing, and Alignment 

Total RNA from seed samples was extracted with a Trizol reagent kit (Invitrogen, Carlsbad, CA, USA) as per the manufacturer’s specifications. The genomic DNA was discarded using DNase I (TaKara, Beijing, China). RNA quality was investigated on an Agilent 2100 Bioanalyzer (Agilent Technologies, Palo Alto, CA, USA) and quantified using the ND-2000 (NanoDrop Technologies). Only high-quality RNA (OD260/280 = 1.8~2.2, OD260/230 ≥ 2.0, RIN ≥ 6.5, 28S:18S ≥ 1.0) samples were used for sequencing library construction using a TruSeqTM RNA sample preparation Kit (Illumina, San Diego, CA, USA). After qualified mRNA fragmentation, cDNAs were constructed using NEB (Next Ultra RNA Library Prep Kit, Ipswich, MA, USA) and adapters were ligated. The resulting cDNA library was sequenced on the Illumina sequencing platform (HiSeq xten/NovaSeq6000 sequencer). SeqPrep (https://github.com/jstjohn/SeqPrep; access on 1 April 2024) and Sickle (https://github.com/najoshi/sickle; access on 15 March 2024) software were used to check the quality of raw paired ends. The clean reads were aligned to the sesame reference genome [38] by the HISAT2 (http://ccb.jhu.edu/software/hisat2/index.shtml; access on 10 March 2024) software [39]. Finally, we assembled the mapped reads using StringTie (http://www.string-db.org/; access on 18 March 2024) [40].

### 2.7. Differentially Expressed Genes (DEGs) and Functional Enrichment Analysis

The expression level of transcripts was computed according to the transcripts per million reads (TPM) method and RSEM (http://deweylab.biostat.wisc.edu/rsem/; access on 11 March 2024) was used to quantify each gene abundance [41]. DEG analysis was carried out using the DESeq2 software [42] at FDR (false discovery rate) ˂ 0.05 and |fold change| ≥ 1. KEGG (Kyoto Encyclopedia of Genes and Genomes, http://www.genome.jp/kegg/kaas; access on 11 March 2024) and GO (Genes Ontology, http://geneontology.org/; access on 11 March 2024) enrichment analyses were achieved using KOBAS (version 3.0) and GO seq package in R (version 4.3), respectively. Significant enrichment terms were screened out at *p*-value < 0.05.

### 2.8. Quantitative RT–PCR Analysis

The RNA was extracted from developing seed samples using a modified CTAB method [43]. Reverse transcription (RT) was conducted with the Monad 1st Strand cDNA Synthesis Kit and the qRT-PCR analysis was achieved using Tb Green^®^ Premix Ex Taq™ II (Takara, Beijing, China) as previously described [44]. All samples had three biological and technical replicates. The sesame histone gene (*SiH3.3*) was used as an internal control to normalize the expression levels of target genes via the 2^−ΔΔCT^ method [45]. The NCBI’s primer designing tool, PRIMER-BLAST (Primer3), was used to design specific primers for each gene (Table S7).

## 3. Results and Discussion

### 3.1. Variation in Antioxidant Activity (AOA), Total Flavonoid (TFC), and Phenolic (TPC) Contents in the Sesame Population 

In order to thoroughly examine the variability of sesame seeds’ antioxidant capability, we evaluated the AOA, TPC, and TFC of seeds from 400 diverse sesame accessions (Table S1). The frequency distribution of the three traits in the population is presented in Figure S1. In general, the AOA of the seeds varied from 2.03 to 78.5%, with a CV (coefficient of variation) of 45.12% (Table 1). Meanwhile, the TPC ranged from 2.717 to 21.98 mg GAE/g, with a CV of 44.52% (Table 1). The TFC varied from 0.072 to 3.104 mg CAE/g, with a CV of 42.29% (Table 1). These results show a significant variation in sesame seed antioxidant capacity and polyphenol profiles driven mainly by the genotypes. 

### 3.2. Correlation between Seed Antioxidant Activity and Seed Phytochemicals 

**Figure 1:**
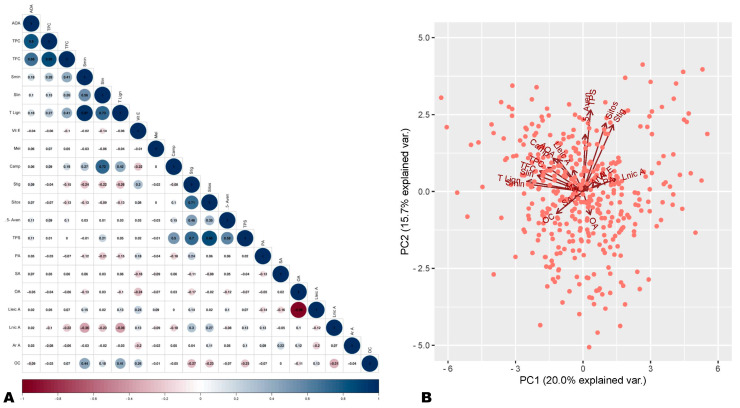
Correlation and principal component analysis of antioxidant activity (AOA) and seed phytochemical components. (**A**) Correlation plot of AOA with seed phytochemical components. (**B**) Principal component analysis plot. AOA, antioxidant activity; TPC, total phenolic content; TFC, total flavonoid content; Smin, sesamin; Slin, sesamolin; T lign, total lignan; Camp, campesterol; 5-Aven, 5-avenasterol; Sitos, sitosterol; Stig, stigmasterol; TPS, total sterols; Vit E, vitamin E (tocopherol); OC, oil content; OA, oleic acid; Lnic A, linolenic acid; SA, stearic acid; Lneic A, linoleic acid; PA, palmitic acid; Mel, melatonin.

To identify the major seed phytochemicals that govern the variation in antioxidant capability, we carried out a correlation analysis. The same seeds were previously analyzed for fatty acid composition and oil content, sesamin and sesamolin (lignans) content, phytosterols content, melatonin content, and tocopherol (Vitamin E) content [44,46,47,48]. These previous data were taken into account for the correlation analysis. The analysis revealed a significantly high positive correlation between AOA and TPC (r = 0.8) and between AOA and TFC (r = 0.66) (Figure 1A and Figure S2). The AOA showed significant low positive correlations with sesamin content (r = 0.18), total lignan (r = 0.18), and total phytosterol (r = 0.11) (Figure 1A and Figure S2). There was a significant negative correlation between AOA and oil content and no significant correlations with other seed components (Figure 1A and Figure S2). These results show that polyphenols are the main antioxidants in sesame seeds. Moreover, they indicate that lignans are not the sole major antioxidants in sesame seeds and that flavonoids, phenolic acids, and other phenolic compounds also significantly influence sesame seed antioxidant capability. Plant polyphenols, including flavonoids and non-flavonoids (stilbenes, phenolic acids, lignans, tannins, etc.), are important antioxidants with high pharmacological values [19,20,49].

We further performed PCA analysis to verify the correlation analysis results. As shown in Figure 1B, the PCA analysis results were supportive of observed correlations. The AOA and polyphenol components (TPC, TFC, sesamin, sesamolin, and total lignan) were projected closely on the PCA plot (Figure 1B). AOA and oil content were projected in opposite directions, confirming their negative correlations (Figure 1B). Overall, these findings denote that variation in polyphenol profiles of sesame seeds may be the key underlying factor of difference in seeds’ antioxidant capabilities. In addition to its inherent lignans, sesame seeds may contain diverse other phenolic compounds with important antioxidant power.

### 3.3. Influence of Seed Coat Colors on Sesame Seed Antioxidant Activity 

**Figure 2:**
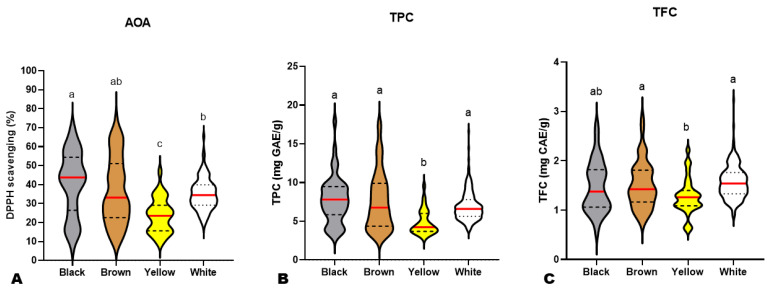
Variation in antioxidant activity (**A**), total phenolic content (**B**), and total flavonoid content (**C**) among black (n = 45), brown (n = 85), yellow (n = 32), and white (n = 234) sesame seeds from different accessions. The red lines indicate the mean. Black dotted/dashed lines indicate quartiles. Different letters above the violin plots indicate statistical differences at *p* ˂ 0.05.

Previous investigations on small numbers of sesame varieties revealed that black seeds possess higher antioxidant capability than other colored sesame seeds [10,13,14,50]. To verify these reports, we compared the AOA, TFC, and TPC of black (BkS), yellow (YS), brown (BnS), and white (WS) sesame seeds (Figure 2). As shown in Figure 2A, most BkS had higher AOA than the majority of other colored seeds. However, the AOA of BkS and BnS were not statistically different (Figure 2A). The AOA of YS was significantly the lowest (Figure 2A). Although white seeds showed significantly lower AOA than black seeds and similarity to brown seeds, the lowest AOA values were recorded on some dark (black and brown) accessions (Figure 2A). These results denote that the AOA of sesame seeds is a complex trait, irrespective of seed coat color. Not all dark sesame seeds may possess high antioxidant capability. It is therefore required to dissect the molecular network regulating the sesame seed antioxidants for exploitation in developing novel varieties with improved antioxidant capability.

Regarding the TPC and TFC, the white, brown, and black seeds exhibited statistically similar results (Figure 2B,C). The yellow seeds had the lowest TPC and TFC, as per the AOA (Figure 2B,C). Taken together, these findings infer that the antioxidant capacity of sesame seeds varies mostly upon the polyphenol profile and the variation characteristics of each phenolic compound in the different colored seeds.

### 3.4. Polyphenol Profiles of High and Low Antioxidant Sesame Seeds 

**Figure 3:**
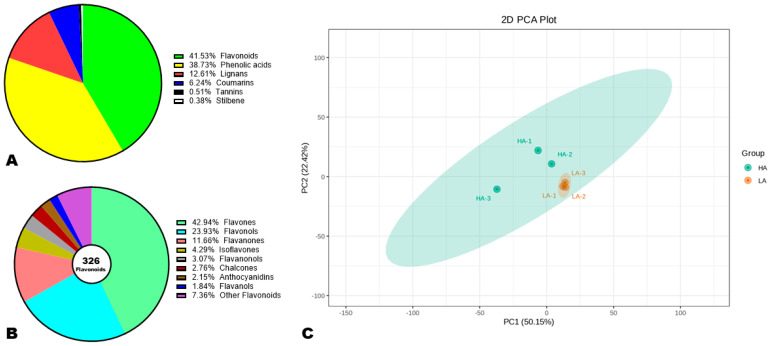
Variation in the polyphenol profiles of high (HA) and low (LA) antioxidant seeds. (**A**) Classification of the 785 identified phenolic compounds. (**B**) Sub-classification of flavonoids. (**C**) Principal component analysis (PCA). HA-1, HA-2, and HA-3 represent the three replications for HA. LA-1, LA-2, and LA-3 represent the three replications for LA.

To reveal the major phenolic compounds responsible for variation in sesame seeds’ AOA, we performed comparative widely targeted polyphenol profiling of high-antioxidant (HA) and low-antioxidant (LA) accessions [34,36]. As shown in Figure S3, the detection of the metabolites was achieved in both electrospray ionization modes. The repeatability of the experiment was confirmed by the high correlations (r ≥ 0.99) recorded between QC samples (Figure S4). We structurally identified a total of 785 phenolic compounds in sesame seeds, including 41.53% flavonoids, 38.78% phenolic acids, 12.61% lignans, 6.24% coumarins, 0.51% tannins, and 0.38% stilbenes (Figure 3A, Table S3). This result shows that flavonoids and phenolic acids are the foremost phenolic compounds in sesame seeds. Accordingly, they may have a greater influence on seed AOA than lignans. It is reported that lignans are the primary antioxidant compounds in sesame seeds [5,51]. Of the 326 identified flavonoids, flavones (42.94%), flavonols (23.93%), and flavanones (11.66%) were dominant (Figure 3B). Isoflavones and anthocyanidins accounted for 4.29 and 2.15%, respectively (Figure 3B).

To explore the variability in metabolites between HA and LA seeds, we conducted HCA and PCA analysis (Figure 3C and Figure S5). As shown in Figure S5, the HCA revealed remarkable differences between the polyphenol profiles of HA and LA seeds. The majority of the phenolic compounds showed the highest relative content in HA compared to LA seeds (Figure S5). The PCA confirmed that the polyphenol profiles of HA and LA seeds were very different and could be discriminated by PC1 (50.15%) and PC2 (22.42%) (Figure 3C). These results represent support for the correlation between sesame seed AOA and polyphenol profile.

### 3.5. Differentially Accumulated Phenolic (DAPs) Compounds and KEGG Analysis 

**Figure 4:**
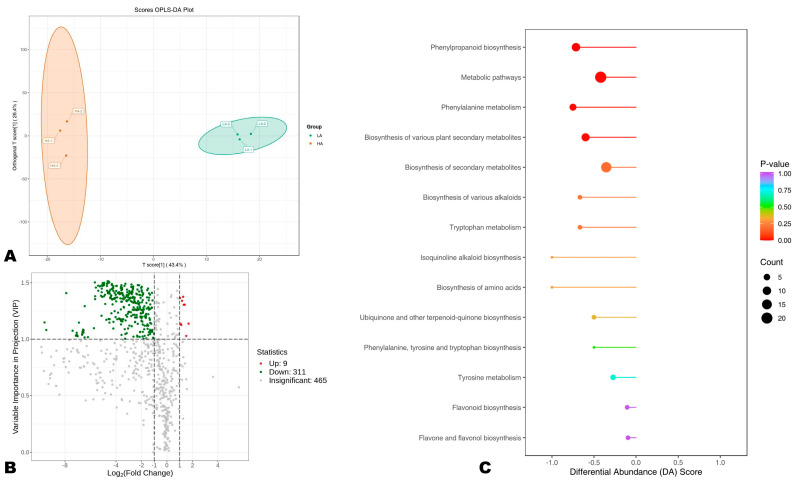
Differentially accumulated phenolic (DAP) compounds between high (HA) and low (LA) antioxidant seeds. (**A**) Score plot of the OPLS-DA analysis. (**B**) Volcano plot of the DAPs between HA and LA. Up-regulation and down-regulation indicate the metabolite has higher relative content in LA and HA, respectively. (**C**) KEGG annotation and enrichment results of DAPs.

In order to uncover DAPs between HA and LA sesame seeds, we carried out OPLS-DA analysis. The score plot of the OPLS-DA confirmed the great difference in the polyphenol profiles between the two groups (Figure 4A). The R^2^Y and Q^2^ of the pairwise comparison were 0.997 and 0.874, respectively, indicating the reliability of the model (Figure S6). We uncovered a total of 320 DAPs, including 311 highly accumulated in HA compared to LA seeds (Figure 4B, Table S4). The DAPs included 145 phenolic acids, 87 flavonoids, 64 lignans, 23 coumarins, and 1 tannin. Sesamolinol-glucoside and sesamolinol 4′-*O*-β-D-glucosyl (1→6)-*O*-β-D-glucoside were only two differentially accumulated sesame-specific lignans, supporting that the variation in seeds’ AOA could not be attributed to differences in the content of specific lignans, such as sesamin, sesamolin, sesamolinol, etc., only. Phenolic acids, flavonoids, and other lignans are critical for the high AOA of sesame seeds.

To provide insights into differential molecular mechanisms between HA and LA seeds, we performed a KEEG analysis of DAPs (Figure 4C). The results showed that the main pathways differentially regulated between HA and LA were phenylalanine metabolism, biosynthesis of secondary metabolites, flavonoid biosynthesis, phenylpropanoid biosynthesis, and tyrosine metabolism (Figure 4C). Phenolic compounds are synthesized in plants from phenylalanine, tyrosine, and tryptophan, themselves occurring from chorismate (the ultimate product of the shikimate pathway) [52,53]. Collectively, these findings infer that the antioxidant capacity of sesame seeds may be improved by inducing phenylalanine biosynthesis, phenylpropanoid biosynthesis, and flavonoid accumulation in developing seeds [54,55]. Investigating gene-metabolite interactions in these pathways may offer crucial genetic resources for improving sesame seed antioxidant capability.

### 3.6. Major Highly Accumulated Phenolic Compounds in High-Antioxidant Sesame Seeds

To reveal the major highly accumulated phenolic compounds in HA seeds, we filtered out the top 50 up-regulated metabolites in HA (Table 2). The top 50 up-regulated DAPs in HA included 29 flavonoids, 14 phenolic acids, 5 lignans, and 2 coumarins. It was worth noting that the top 20 highly accumulated phenolic compounds in HA seeds were all flavonoids (Figure S7). These major up-regulated DAPs in HA merit being investigated in future studies to better understand sesame seed bioactivities. For instance, tricin, the top DAP (|Log2FC| = 9.593), possesses diverse therapeutical potentials, including anti-cancer, anti-influenza, anti-angiogenic, and antioxidant effects [56,57,58]. Diosmetin, peonidin, and apigenin have also recorded pharmacological attributes, such as anti-cancer, antioxidant, neuroprotective, etc. [59,60,61]. Matairesinol has demonstrated antioxidant, anti-cancer, neuroprotective, and anti-inflammation abilities [62,63]. In addition, the major DAPs could serve as key biomarkers for analyzing molecular networks regulating polyphenol biosynthesis during sesame seed development. As a support, correlation network analysis among DAPs revealed significant positive correlations between 22 phenolic acids, 17 lignans, and 10 flavonoids (Figure S8).

### 3.7. Differentially Expressed Genes (DEGs) between HA and LA during Seed Development

**Figure 5:**
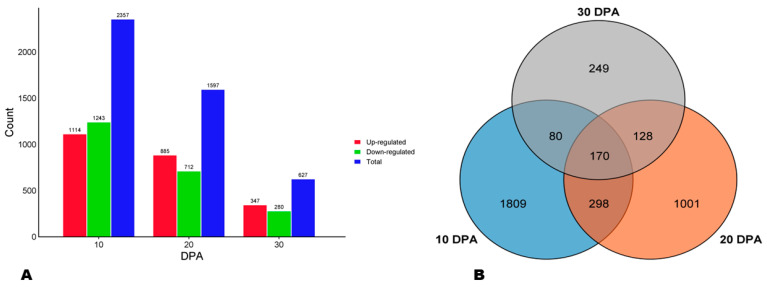
Differentially expressed genes (DEGs) between HA and LA. (**A**) Number of DEGs at the three seed developmental stages. (**B**) Venn diagram indicating the number of key DEGs.

To verify the implication of phenylpropanoid metabolism in variation in sesame seeds AOA, we carried out a comparative transcriptome analysis of HA and LA varieties at three seed developmental stages, including 10, 20, and 30 DPA (days post-anthesis). The summary of the high-throughput RNA sequencing data is presented in Table S5. The reliability of the RNA-seq data was confirmed through qRT-PCR analysis of eight randomly selected genes, with a consistency of R^2^ of 0.91 (Figure S9). Analyses revealed 2357, 1597, and 627 DEGs between HA and LA at 10, 20, and 30 DPA, respectively (Figure 5A). Of these DEGs, 1114, 885, and 347 were up-regulated in HA at the respective developmental stages (Figure 5A). A Venn diagram showed that only 170 genes were differentially expressed between the two seed types along with the seed development (Figure 5B).

**Figure 6:**
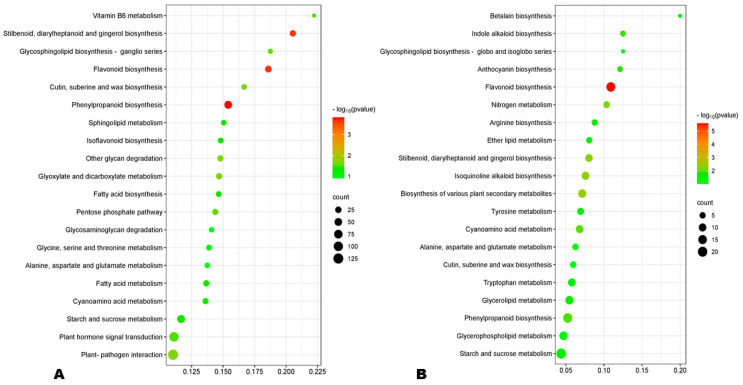
(**A**,**B**) KEGG enrichment of DEGs between HA and LA at 10 and 30 DPA (days post-anthesis), respectively.

GO (gene ontology) analysis revealed that the DEGs at 10 DPA were mostly enriched to the membrane and its components, oxidoreductase activity, lipid storage, and carbohydrate metabolic process (Figure S10A). Meanwhile, the main enriched GO terms at 20 DPA were intracellular non-membrane, ribosome, structural constituent of ribosome, structural molecule activity, and peptide and amide metabolic processes (Figure S10B). At 30 DPA, the most enriched GO terms were extracellular region, carbohydrate metabolic process, and iron ion binding (Figure S11). These results indicate different metabolism regulations during HA and LA seed developmental processes. KEGG enrichment analysis of DEGs revealed that phenylpropanoid biosynthesis, flavonoid biosynthesis, and stilbenoid biosynthesis were the most significantly differentially regulated pathways between HA and LA at early and late seed developmental stages (Figure 6A,B). Meanwhile, ribosome metabolic processes were the main processes differentially regulated at 20 DPA (Figure S12). Taken together, these results show that differences in the regulation of flavonoid and phenolic acid biosynthesis are the key driven mechanisms of variation in sesame seed AOA.

### 3.8. Expression Patterns of Phenylpropanoid Biosynthesis-Related DEGs

**Figure 7:**
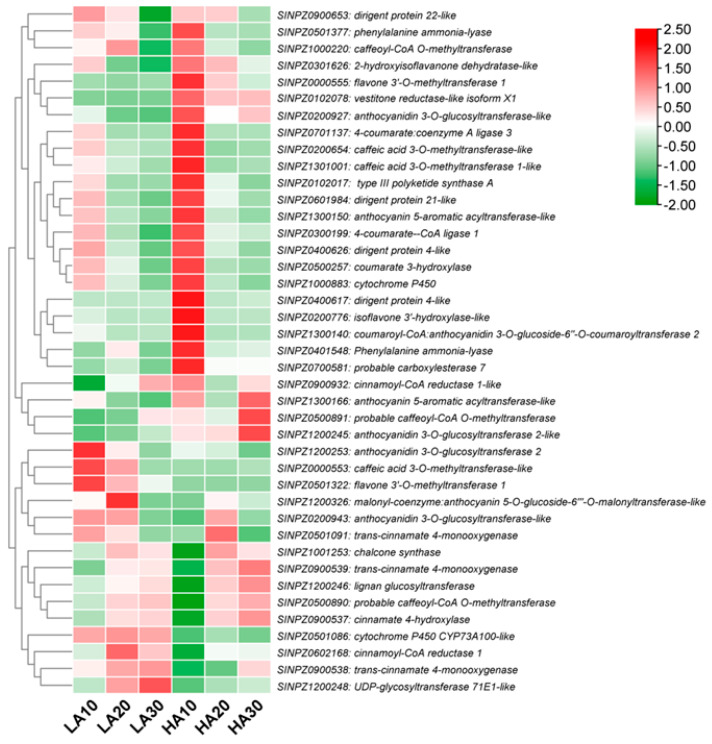
Expression patterns of phenylpropanoid pathway-related DEGs in HA and LA during seed development. The values 10, 20, and 30 indicate DPA.

Based on the above results, we found it important to examine the expression patterns of phenylpropanoid pathway-related DEGs to identify potential target genes for modulating the sesame polyphenol profile. As shown in Figure 7, most phenylpropanoid structural genes, such as phenylalanine ammonia-lyase (*SINPZ0401548* and *SINPZ0501377*), caffeoyl-CoA O-methyltransferase (*SINPZ1000220* and *SINPZ0500891*), caffeic acid 3-O-methyltransferase (*SINPZ0200654* and *SINPZ1301001*), coumarate 3-hydroxylase (*SINPZ0500257*), isoflavone 3′-hydroxylase (*SINPZ0200776*), cinnamoyl-CoA reductase (*SINPZ0900932*), vestitone reductase (*SINPZ0102078*), etc., were up-regulated in HA, particularly at 10 DPA. Meanwhile, the main up-regulated genes in LA included cinnamoyl-CoA reductase 1 (*SINPZ0602168*), trans-cinnamate 4-monooxygenase (*SINPZ0900538* and *SINPZ0501091*), UDP-glycosyltransferase 71E1 (*SINPZ1200248*), cytochrome P450 CYP73A100 (*SINPZ0501086*), caffeic acid 3-O-methyltransferase (*SINPZ0000553*), anthocyanidin 3-O-glucosyltransferase 2 (*SINPZ1200253*), and flavone 3′-O-methyltransferase 1 (*SINPZ0501322*) (Figure 7). These genes represent important resources for quality improvement in sesame.

### 3.9. Key Differentially Expressed Transcription Factors (TFs)

**Figure 8:**
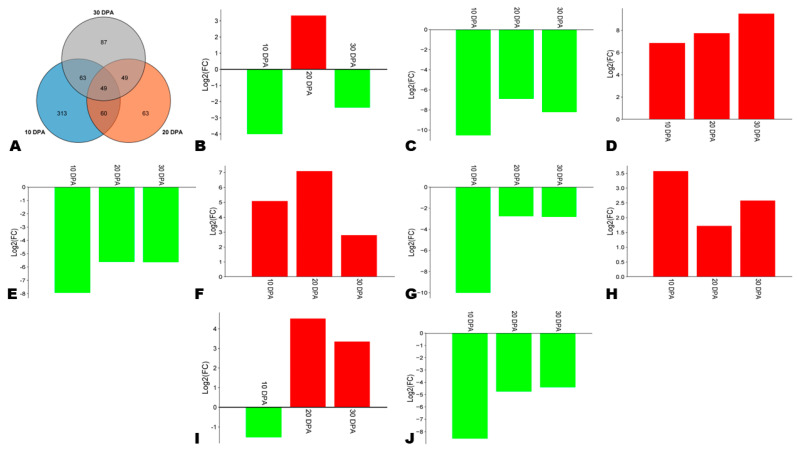
Key differentially regulated transcription factors (TFs) between HA and LA. (**A**) Venn diagram indicating the number of key TFs. (**B**–**J**) Expression fold changes in major differentially regulated TFs, including *SINPZ0900373* (NAC), *SINPZ0801455* (MADS), *SINPZ0500871* (C3H), *SINPZ1300846* (MYB), *SINPZ0000571* (MYB), *SINPZ0100001* (bHLH), *SINPZ0401118* (NAC), *SINPZ0300035* (MYB), and *SINPZ0501303* (Trihelix).

TFs play critical regulatory functions in phenylpropanoid biosynthesis, particularly MYB and NAC [26,64]. We screened out 49 key differentially expressed TFs, including 9 with considerable expression fold changes (Figure 8A, Table S6). Three genes, including *SINPZ0500871* (C3H), *SINPZ0000571* (MYB), and *SINPZ0401118* (NAC), and were highly induced in HA along with the seed development (Figure 8D,F,H). The gene *SINPZ0300035* (MYB) was down-regulated at 10 DPA and subsequently highly up-regulated at 20 and 30 DPA in HA (Figure 8I). The NAC gene *SINPZ0900373* was down-regulated in HA except at 20 DPA (Figure 8B). Four genes, including *SINPZ0801455* (MADS), *SINPZ1300846* (MYB), *SINPZ0100001* (bHLH), and *SINPZ0501303* (Trihelix), were highly induced in LA compared to HA (Figure 8C,E,G,J). These genes need to be functionally characterized for a deep understanding of the regulatory network of phenolic compounds’ biosynthesis and accumulation in sesame seeds.

## 4. Conclusions

In summary, this study offers an understanding of the biochemical and molecular basis of variation in sesame seeds’ antioxidant capability through integrated phytochemical analysis, polyphenol profiling, and transcriptome sequencing. It revealed a significant variation in seeds’ AOA, TPC, and TFC in a panel of 400 sesame accessions. Analyses showed that although sesame-specific lignans have very high AOA, they contribute less to the differences in the AOA of seeds from different genotypes. Differences in phenolic acid and flavonoid profiles are the prime contributors to seed antioxidant capacity variation, irrespective of seed coat color. Other seed components, such as fatty acids, melatonin, tocopherol, etc., have no significant correlations with AOA. In total, 311 highly accumulated phenolic compounds in HA seeds were identified. It was worth noting that the top 20 up-regulated DAPs in HA were all flavonoids. DEGs between HA and LA were identified and functionally annotated. The key molecular mechanisms governing the variation in seed AOA were flavonoid biosynthesis, phenylpropanoid biosynthesis, and stilbene biosynthesis. Furthermore, key differentially regulated phenylpropanoid structural genes and candidate TF genes were filtered out. Our findings bring to light key mechanisms and sesame seed antioxidants driving the variation in seed antioxidant capacity. Moreover, they offer fundamental resources for improving sesame’s medicinal value.

## Figures and Tables

**Table 1:** Variation in antioxidant activity, total phenolic content, and total flavonoid content in 400 sesame seed accessions.

Traits	Min	Max	Mean	SD	SE	CV (%)	Skewness	Kurtosis
AOA (%)	2.035	78.5	33.57	15.15	0.757	45.12	0.506	0.054
TPC (mg GAE/g)	2.717	21.98	6.9	3.072	0.154	44.52	1.478	3.415
TFC (mg CAE/g)	0.072	3.104	1.255	0.531	0.027	42.29	0.418	0.461

AOA, antioxidant activity (DPPH scavenging); TPC, total phenolic content; TFC, total flavonoid content; Min, minimum; Max, maximum; SD, standard deviation; SE, standard error; CV, coefficient of variation; GAE, gallic acid equivalent; CAE, catechin equivalent.

**Table 2:** List of the 50 top up-regulated phenolic compounds in high-antioxidant sesame seeds.

Categories	Sub-Class	Compounds	Formula	VIP	*p*-Value	FDR	|Log2FC|
Flavonoids	Flavones	Tricin (5,7,4′-Trihydroxy-3′,5′-dimethoxyflavone)	C_17_H_14_O_7_	1.149	0.413	0.548	9.593
	Flavanones	Persicoside	C_23_H_26_O_11_	1.081	0.419	0.548	9.457
	Flavones	5,7,4′,5′-Tetrahydro-3′,6-dimethoxyflavone	C_17_H_14_O_8_	1.409	0.349	0.548	7.906
	Flavones	8-Methoxyapigenin	C_16_H_12_O_6_	1.075	0.415	0.548	7.409
	Flavones	6,7,8-Tetrahydroxy-5-methoxyflavone	C_16_H_12_O_6_	1.033	0.417	0.548	7.143
	Flavones	5,7,3′,5′-tetrahydroxy-6-methylfavanone	C_16_H_12_O_6_	1.030	0.417	0.548	7.138
	Flavones	Scutevulin	C_16_H_12_O_6_	1.057	0.416	0.548	7.083
	Isoflavones	Tectorigenin	C_16_H_12_O_6_	1.036	0.416	0.548	7.081
	Flavones	5,6,7-Tetrahydroxy-8-methoxyflavone	C_16_H_12_O_6_	1.037	0.416	0.548	7.076
	Isoflavones	5,7,4′-Trihydroxy-3′-methoxyisoflavone; 3′-*O*-methylorobol	C_16_H_12_O_6_	1.028	0.417	0.548	7.022
	Flavones	Rhamnocitrin (7-methylkaempferol)	C_16_H_12_O_6_	1.026	0.417	0.548	7.015
	Flavones	Chrysoeriol-7-*O*-gentiobioside	C_28_H_32_O_16_	1.147	0.405	0.548	6.910
	Anthocyanidins	Peonidin-3-*O*-(6′-*O*-caffeoyl)glucoside	C_31_H_29_O_14+_	1.039	0.411	0.548	6.664
	Flavones	Chrysoeriol-7,4′-di-*O*-glucoside	C_28_H_32_O_16_	1.049	0.412	0.548	6.643
	Other Flavonoids	9,11-dimethoxy-2h-[1,3]dioxolo [4,5-b]xanthen-10-one	C_16_H_12_O_6_	1.066	0.413	0.548	6.631
	Flavones	Gnetifolin B	C_16_H_12_O_6_	1.084	0.412	0.548	6.583
	Isoflavones	Aracarpene 2	C_16_H_12_O_6_	1.075	0.412	0.548	6.560
	Flavones	5,7,2′-Trihydroxy-8-methoxyflavone	C_16_H_12_O_6_	1.080	0.412	0.548	6.553
	Flavones	Diosmetin (5,7,3′-Trihydroxy-4′-methoxyflavone)	C_16_H_12_O_6_	1.060	0.413	0.548	6.553
	Other Flavonoids	3-(3,4-dihydroxybenzyl)-5,7-dihydroxy-6-methoxychroman-4-one diglucoside	C_29_H_36_O_17_	1.015	0.410	0.548	6.465
	Flavones	5,7,3′,4′-Tetrahydroxy-6-methoxyflavone-8-C-[glucosyl-(1-2)]-glucoside	C_28_H_32_O_17_	1.018	0.410	0.548	6.204
	Flavonols	Patuletin-3-*O*-gentiobioside	C_28_H_32_O_18_	1.097	0.397	0.548	5.734
	Flavones	Gardenin B 5-(6′-Malonyl)glucoside	C_28_H_30_O_15_	1.430	0.100	0.542	5.393
	Flavonols	Herbacetin	C_15_H_10_O_7_	1.385	0.244	0.548	5.178
	Flavonols	Azaleatin (5-*O*-methylquercetin)	C_16_H_12_O_7_	1.476	0.113	0.542	5.142
	Flavones	Tricetin-5-*O*-(6′-malonyl)glucoside	C_24_H_22_O_15_	1.417	0.126	0.542	5.123
	Other Flavonoids	2′,7-Dihydroxy-3′,4′-dimethoxyisoflavan	C_17_H_18_O_5_	1.375	0.170	0.542	5.076
	Flavones	Viscumneoside III (Homoeriodictyol-7-*O*-apiosyl-(1-2)-glucopyranoside)	C_27_H_32_O_15_	1.472	0.038	0.542	5.061
	Flavones	6-Hydroxyluteolin	C_15_H_10_O_7_	1.380	0.231	0.548	4.950
Lignans and coumarins	Coumarins	Peucedanol	C_14_H_16_O_5_	1.381	0.318	0.548	5.637
	Lignans	Sanshodiol	C_20_H_22_O_6_	1.475	0.090	0.542	5.400
	Lignans	Matairesinol	C_20_H_22_O_6_	1.469	0.092	0.542	5.268
	Lignans	Erythro-guaiacylglycerol-β-coniferyl ether	C_20_H_24_O_7_	1.449	0.150	0.542	5.119
	Lignans	Lappaol C	C_30_H_34_O_10_	1.479	0.086	0.542	4.945
	Coumarins	Esculin glucoside	C_21_H_26_O_14_	1.172	0.369	0.548	4.757
	Lignans	Sesamolinol 4′-*O*-β-D-glucosyl (1→6)-*O*-β-D-glucoside	C_32_H_40_O_17_	1.430	0.196	0.548	4.703
Phenolic acids	Phenolic acids	2′-Acetylacteoside	C_31_H_38_O_16_	1.293	0.310	0.548	6.452
	Phenolic acids	10-Hydroxymajoroside	C_17_H_24_O_11_	1.504	0.017	0.542	5.658
	Phenolic acids	Echinacoside	C_35_H_46_O_20_	1.356	0.230	0.548	5.642
	Phenolic acids	6-*O*-Feruloyl-β-D-glucose	C_16_H_20_O_9_	1.458	0.124	0.542	5.583
	Phenolic acids	Syringoyl-D-glucose	C_15_H_20_O_10_	1.208	0.185	0.542	5.137
	Phenolic acids	1′-*O*-(3,4-Dihydroxyphenethyl)-*O*-caffeoyl-glucoside	C_23_H_26_O_11_	1.498	0.083	0.542	5.047
	Phenolic acids	Arillatose B	C_22_H_30_O_14_	1.482	0.040	0.542	5.010
	Phenolic acids	5-*O*-β-D-Glucopyranosyl-3-hydrobenzo(b)fu-ran-2-one	C_14_H_16_O_8_	1.486	0.040	0.542	4.954
	Phenolic acids	Rosmarinic acid methyl ester	C_19_H_18_O_8_	1.500	0.043	0.542	4.927
	Phenolic acids	Isoferulic acid	C_10_H_10_O_4_	1.394	0.178	0.542	4.860
	Phenolic acids	Purpureaside C	C_35_H_46_O_20_	1.312	0.255	0.548	4.842
	Phenolic acids	1,7-bis(4-hydroxy-3-methoxyphenyl)hept-1-ene-3-ol	C_21_H_26_O_5_	1.491	0.067	0.542	4.716
	Phenolic acids	Tetrahydrorengyoxide-glucose-caffeoyl	C_23_H_26_O_11_	1.472	0.085	0.542	4.712
	Phenolic acids	3,4,5-Trihydroxy-6-[4-[[(2R)-5-oxooxolan-2-yl]methyl]phenoxy]oxane-2-carboxylic acid	C_17_H_20_O_9_	1.495	0.052	0.542	4.667

Note. VIP, value importance in projection; FDR, false discovery rate; FC, fold change.

## Data Availability

Data will be made available on request.

## Supplementary Materials

The following supporting information can be downloaded at https://www.mdpi.com/article/10.3390/antiox13050514/s1. Figure S1. Frequency distribution of antioxidant activity (A), total phenolic content (B), and total flavonoid content (C) of the 400 seeds from different accessions; Figure S2. Another correlation plot of AOA with seed phytochemical components; Figure S3. Multiple reaction monitoring (MRM) graphs of QC samples showing the total ions current (TIC) of some identified metabolites; Figure S4. Correlation analysis between QC samples; Figure S5. Hierarchical clustering analysis (HCA) of the polyphenol profiles of high (HA) and low (LA) antioxidant seeds; Figure S6. Permutation plot of OPLS-DA results of pairwise comparison between high (HA) and low (LA) antioxidant seeds; Figure S7. Top 20 highly accumulated phenolic compounds in high antioxidant sesame seeds; Figure S8. Correlation analysis network between 49 DAPs; Figure S9. qRT-PCR validation of the RNA-seq data; Figure S10. (A) and (B) GO analysis results of DEGs between LA and HA varieties at 10 and 20DPA, respectively; Figure S11. GO analysis results of DEGs between LA and HA varieties at 30DPA; Figure S12. KEGG analysis results of DEGs between LA and HA varieties at 20DPA; Table S1. List of the 400 sesame accessions analyzed in this study; Table S2. Liquid chromatography and mass spectrometry conditions; Table S3. List of the 785 identified phenolic compounds and their relative contents in high (HA) and low (LA) antioxidant seeds; Table S4. List of the 320 differentially accumulated phenolic compounds; Table S5. Summary of the high-quality transcriptome sequencing data; Table S6. List of the 49 key differentially expressed transcription factor-related genes; Table S7. Primers used for the qRT-PCR.
