# Supplementary material for: Biochemical and Molecular Insights into Variation in Sesame Seed Antioxidant Capability as Revealed by Metabolomics and Transcriptomics Analysis
Source: Antioxidants (Basel). 2024 Apr 25;13(5):514. doi: 10.3390/antiox13050514 (PMC11117558; doi:10.3390/antiox13050514)
Supplement: Supplementary file 1 [file antioxidants-13-00514-s001.zip › Supplementary Figures.pdf]

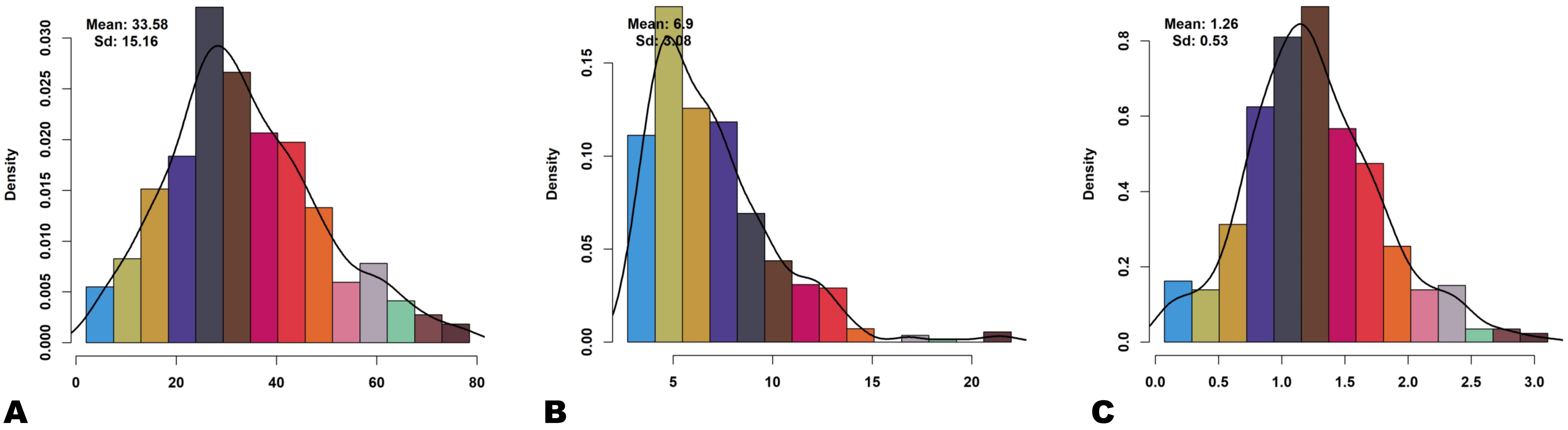

Figure S1. Frequency distribution of antioxidant activity (A), total phenolic content (B), and total flavonoid content (C) of the 400 seeds from different accessions.

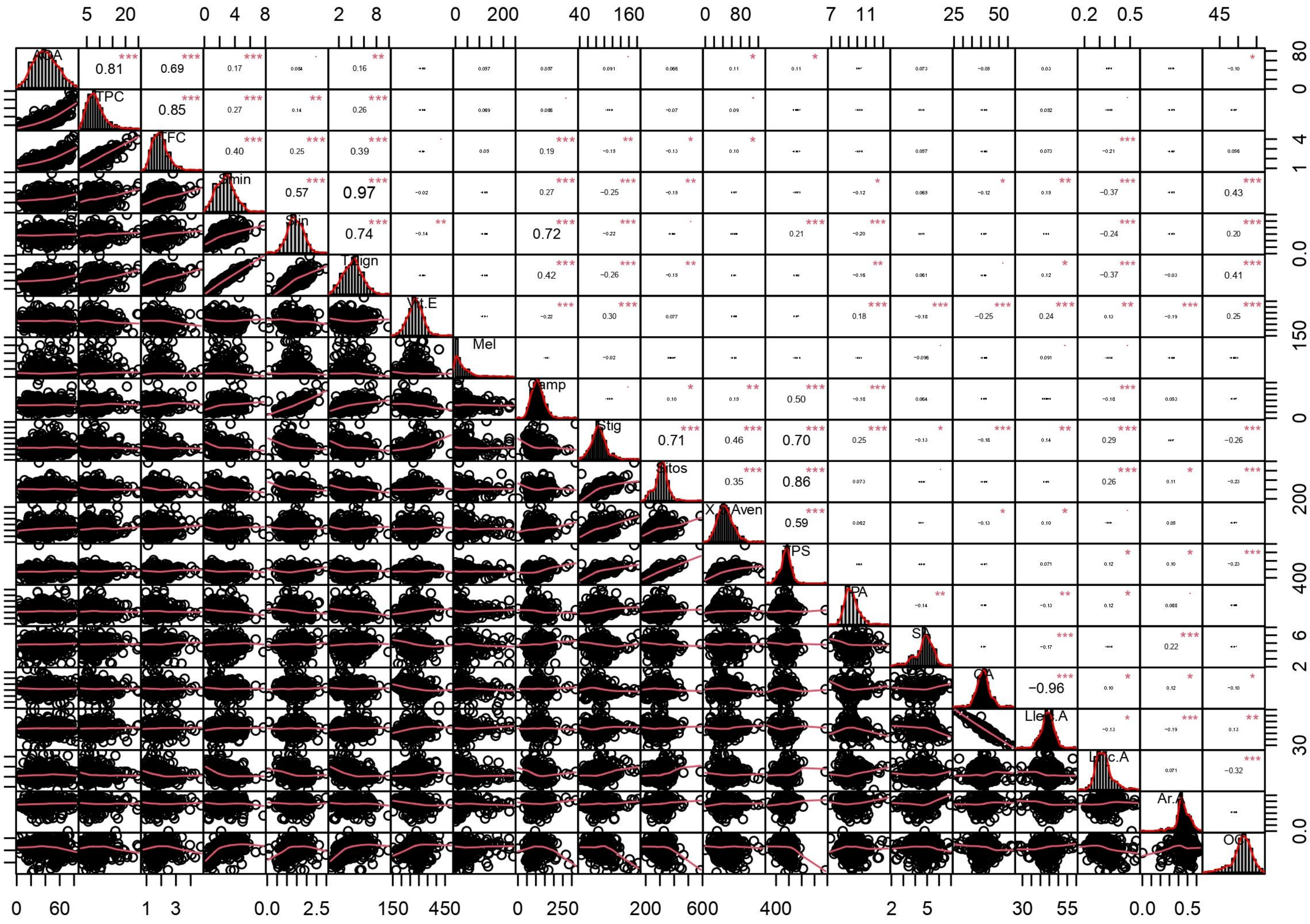

Figure S2. Other correlation plot of AOA with seed phytochemical components.
AOA, antioxidant activity; TPC, total phenolic content; TFC, total flavonoid content; Smin, sesamin; Slin, sesamolin; T lign, total lignan; Camp, campesterol; 5-Aven, 5-avenasterol; Sitos, sitosterol; Stig, stigmasterol; TPS, total sterols; Vit E, vitamin E (tocopherol); OC, oil content; OA, oleic acid; Lnic A, linolenic acid; SA, stearic acid; Lneic A, linoleic acid; PA, palmitic acid; Mel, melatonin. *, **, and *** indicate significant correlations at $P < 0.05$, 0.01, and 0.001, respectively

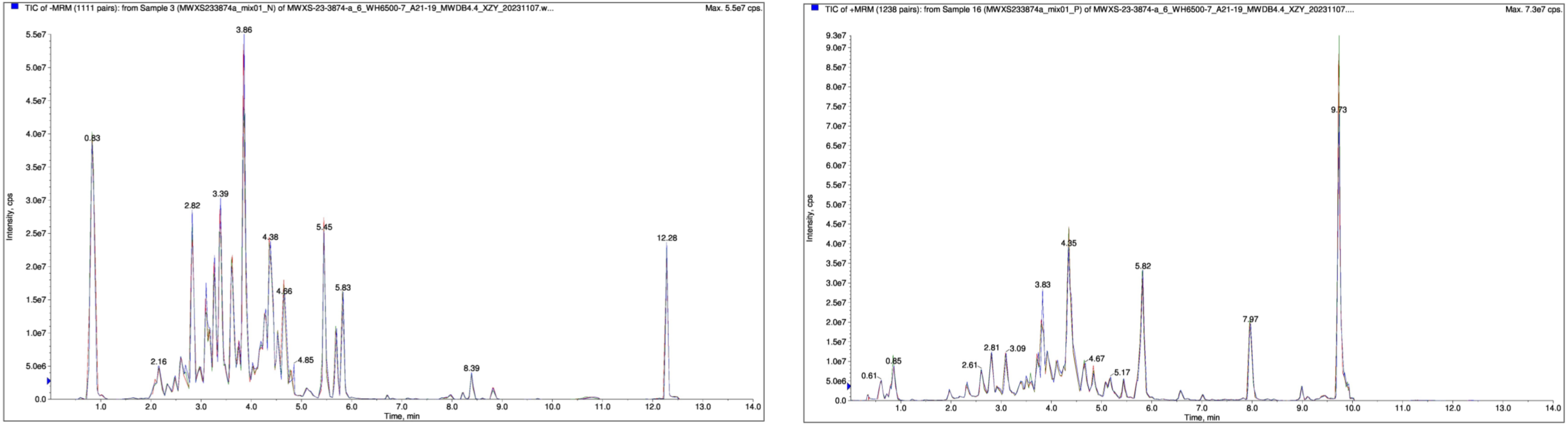

Figure S3. Multiple reaction monitoring (MRM) graphs of QC samples showing the total Ions Current (TIC) of some identified metabolites at the negative and positive ionization, respectively.

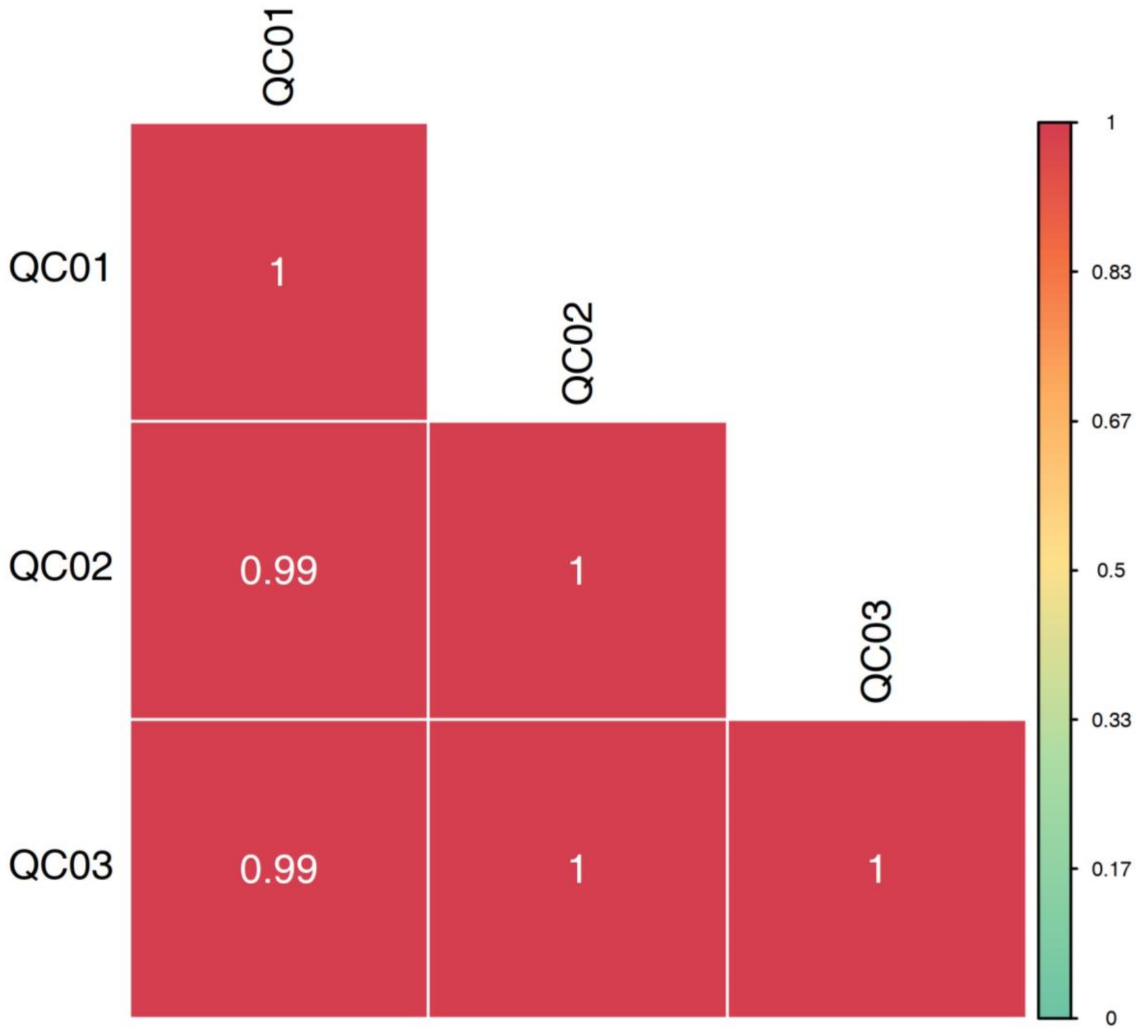

Figure S4. Correlations analysis between QC samples.

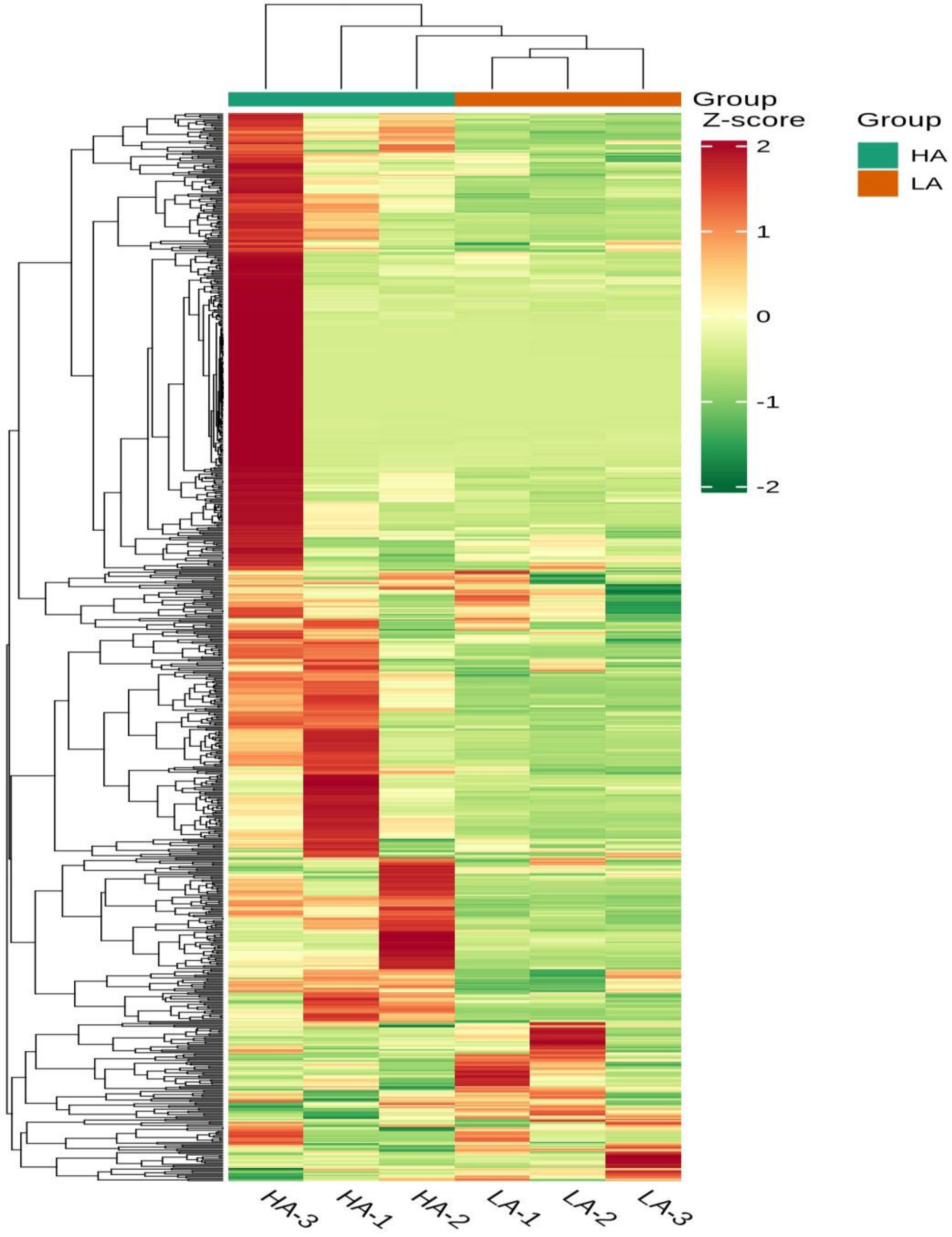

Figure S5. Hierarchical clustering analysis (HCA) of the polyphenol profiles of high (HA) and low (LA) antioxidant seeds.

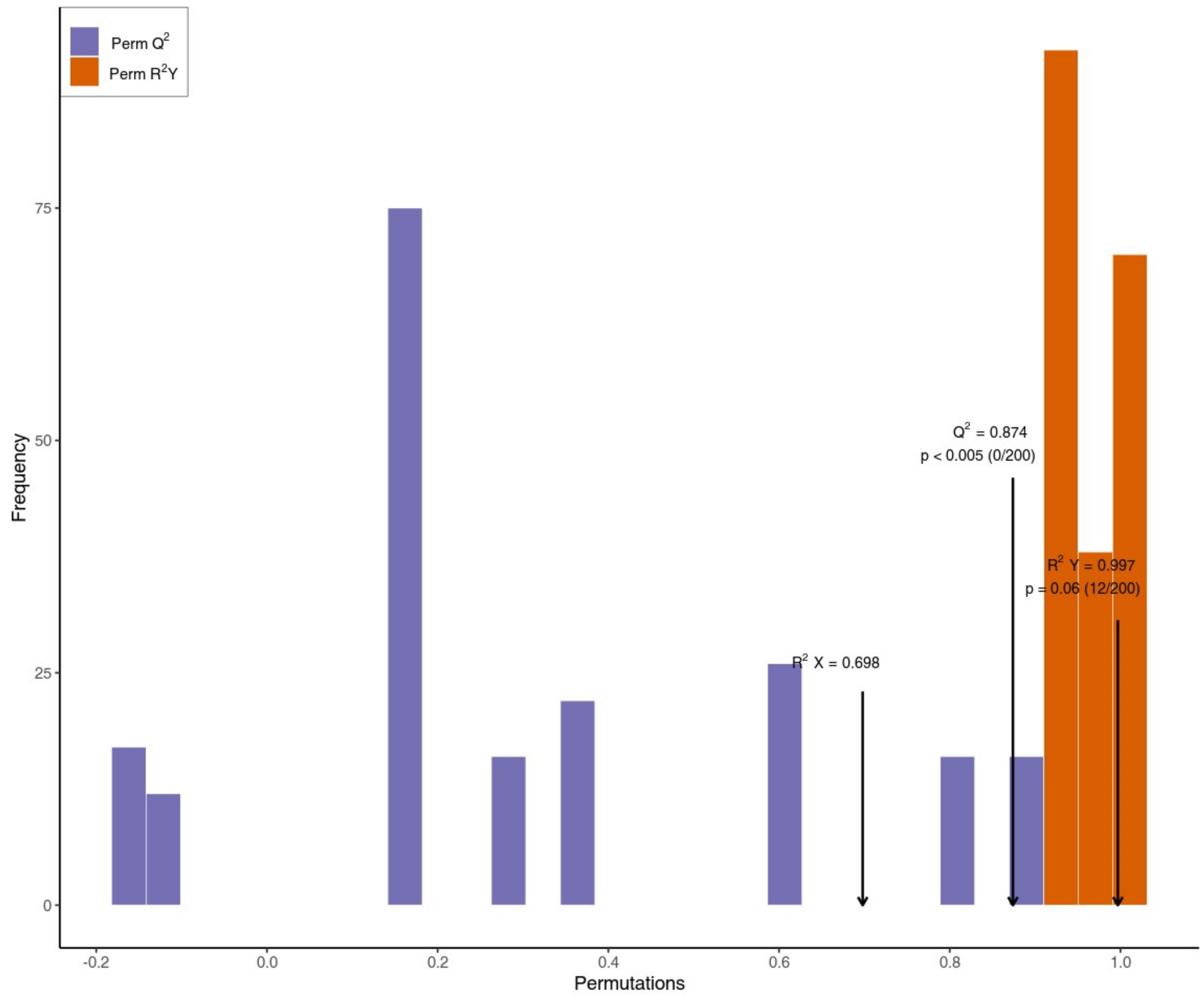

Figure S6. Permutation plot of OPLS-DA results of pairwise comparison between high (HA) and low (LA) antioxidant seeds.

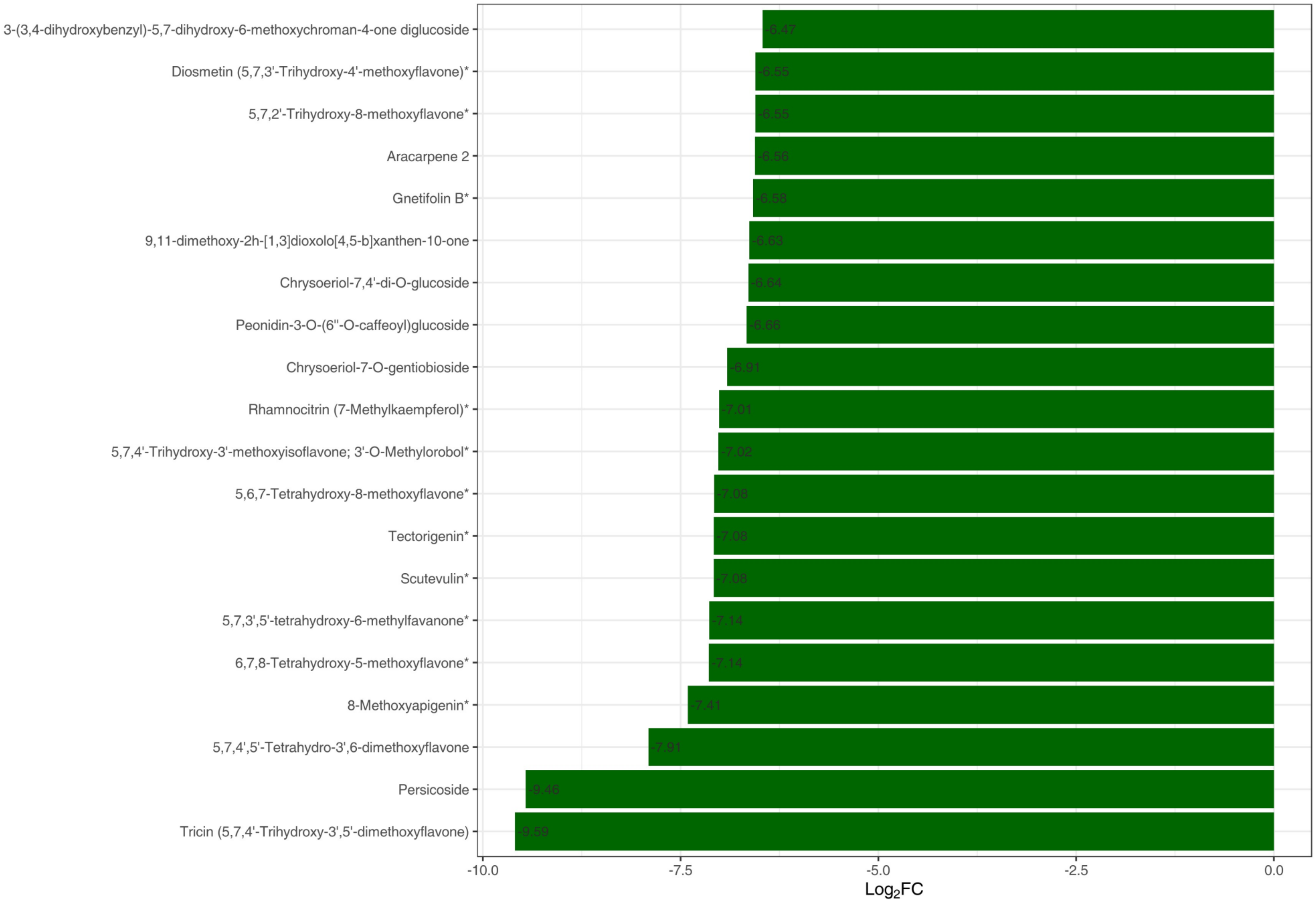

Figure S7. Top 20 highly accumulated phenolic compounds in high antioxidant sesame seeds.

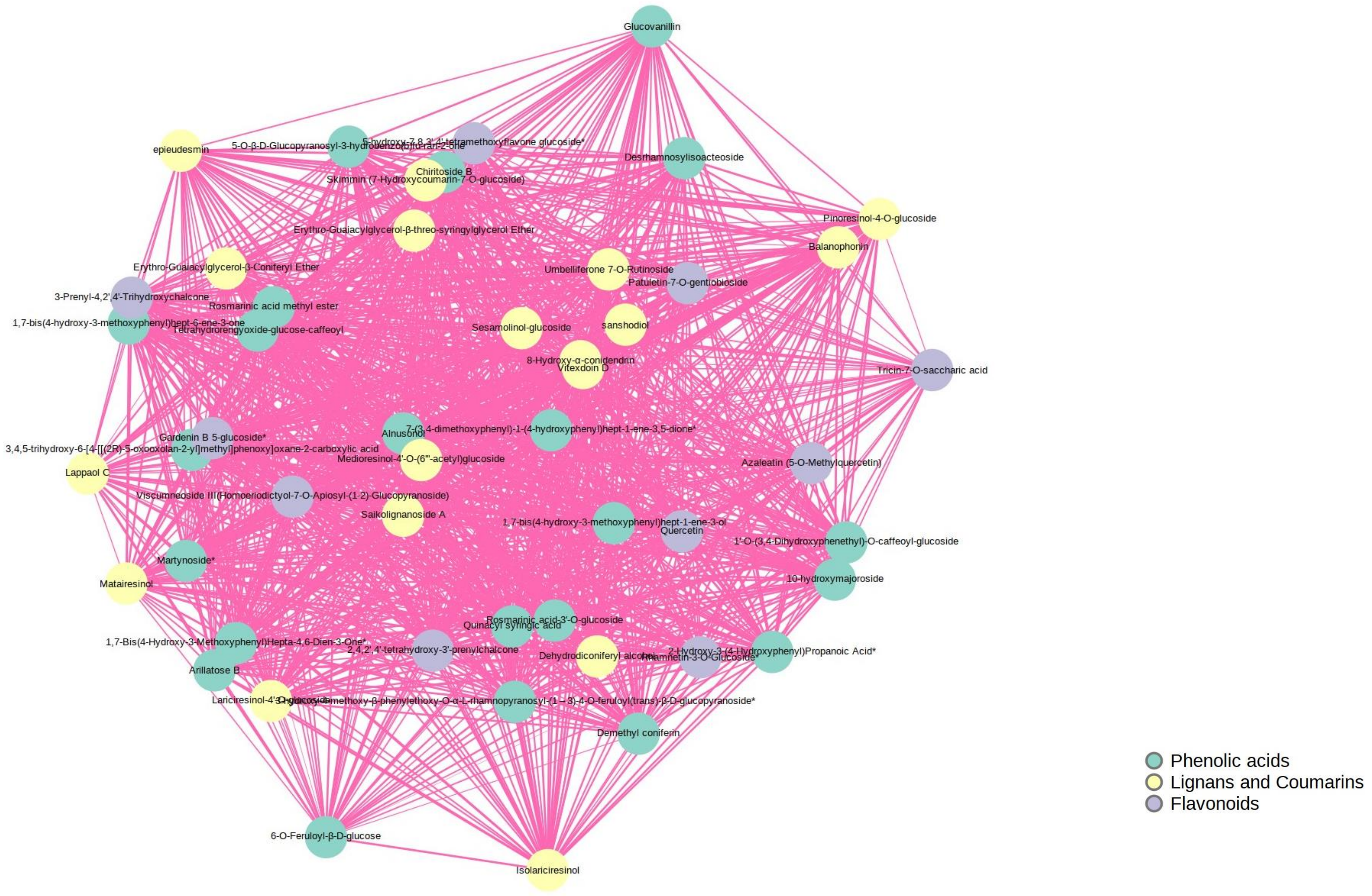

Figure S8. Correlation analysis network between 49 DAPs, including 22 phenolic acids (green circles), 17 lignans (yellow circles), and 10 flavonoids (purple circles). The pink lines indicate higher correlations.

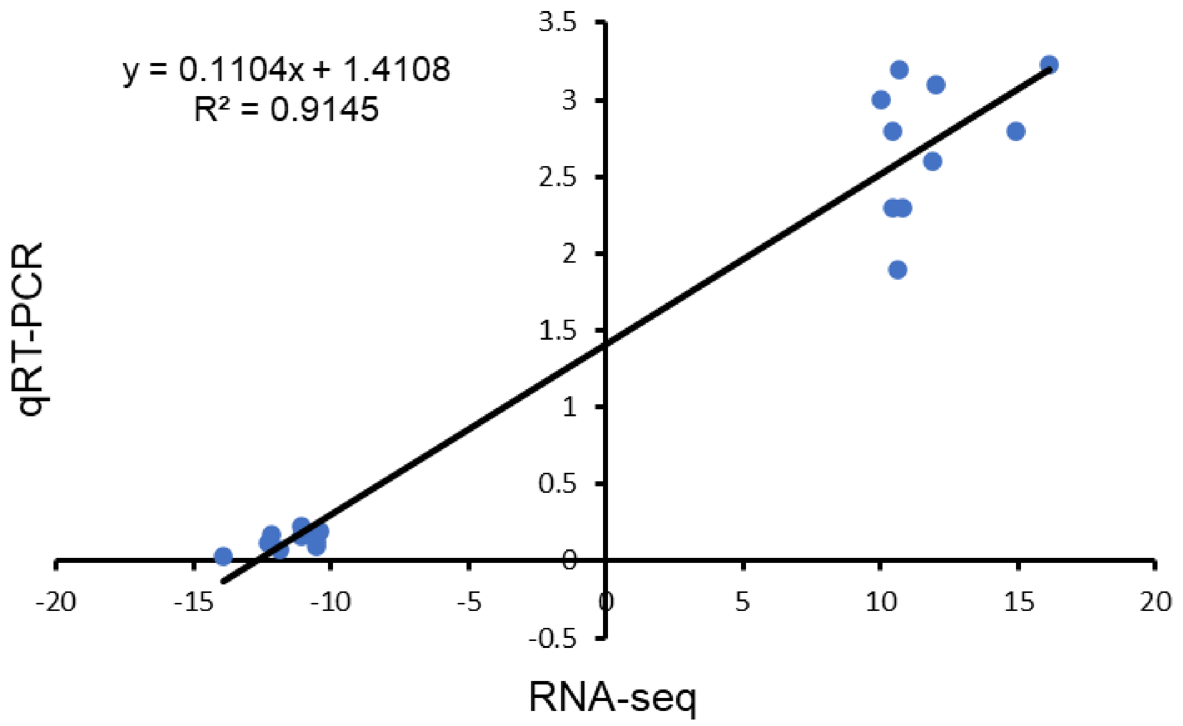

Figure S9. qRT-PCR validation of the RNA-seq data.

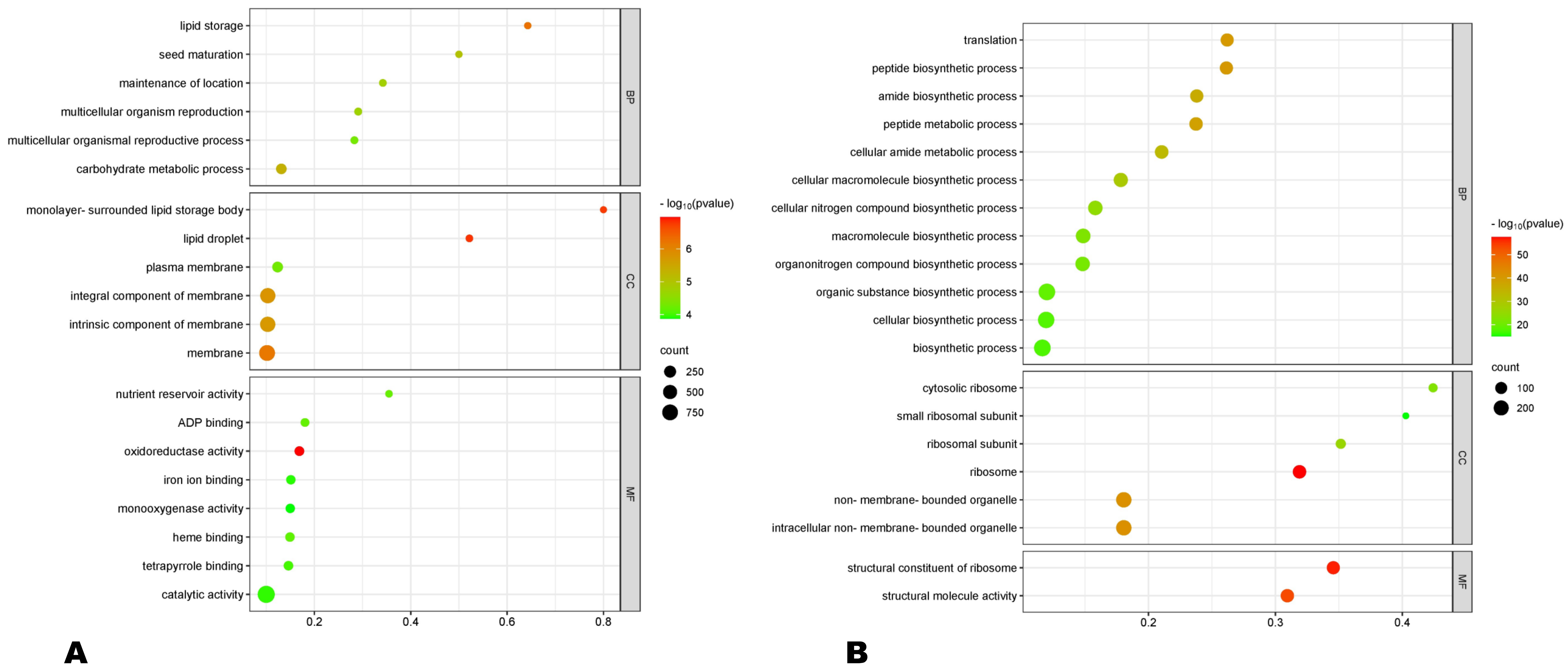

Figure S10. (A) and (B) GO analysis results of DEGs between LA and HA varieties at 10 and 20DPA, respectively.

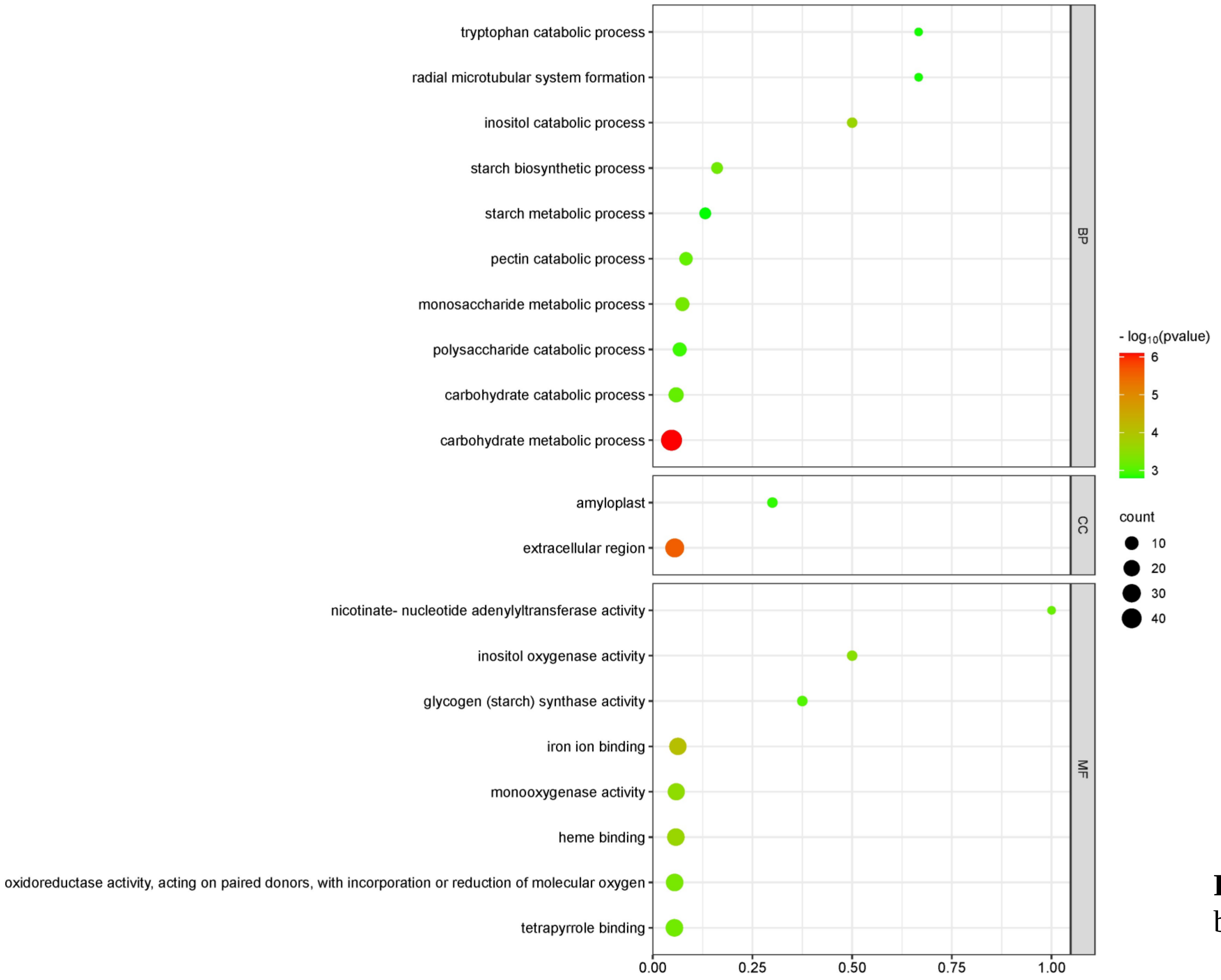

Figure S11. GO analysis results of DEGs between LA and HA varieties at 30DPA.

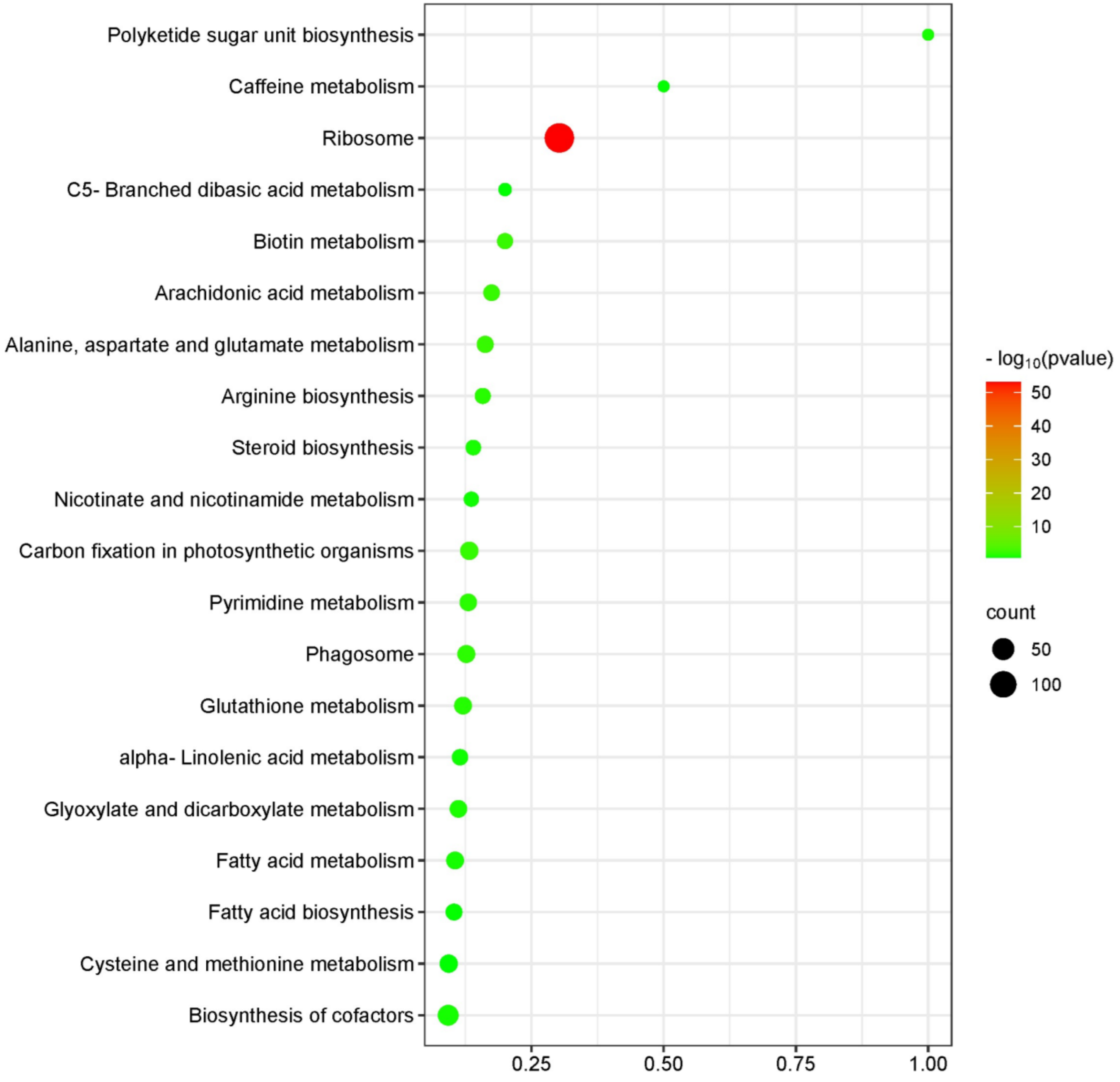

Figure S12. KEGG analysis results of DEGs between LA and HA varieties at 20DPA.
